# Retinal Vascular Density and Vessel Diameter in Sturge–Weber Syndrome Assessed by OCT-Angiography

**DOI:** 10.3390/jcm14197061

**Published:** 2025-10-06

**Authors:** Rosa Longo, Elena Gusson, Erika Lorenzetto, Luca Polinelli, Mariaelena Malvasi, Giacomo Panozzo, Giorgio Marchini

**Affiliations:** 1 Department of Surgery, Dentistry, Paediatrics and Gynaecology, Ophthalmology Clinic, University of Verona, 37126 Verona, Italy; 2 Technology Platform Center, University of Verona, 37126 Verona, Italy; 3 Department of Sense Organs, Faculty of Medicine and Dentistry, Sapienza University of Rome, 00185 Rome, Italy; 4 ESASO, European School of Advanced Studies in Ophthalmology, CH-690 Lugano, Switzerland

**Keywords:** optical coherence tomography, Sturge–Weber syndrome, vascular malformations, OCT angiography, multimodal imaging

## Abstract

**Background:** Sturge–Weber syndrome (SWS) typically presents with a port-wine stain on the face, accompanied by leptomeningeal capillary malformations and ocular vascular anomalies. The aim of our study was to evaluate retinal vascular density and vessel diameter to better characterize the presence of vascular alterations. **Methods:** 15 patients diagnosed with Sturge–Weber syndrome and 15 healthy controls underwent comprehensive ophthalmologic evaluation, Optical Coherence Tomography (OCT) and Optical Coherence Tomography Angiography (OCTA), to evaluate the microvascular architecture of the retina and choroid. **Results:** Analysis of the processed images revealed a significant increase (*p* < 0.05 *) in the density of the deep capillary plexus in patients with SWS compared to healthy controls. Vascular diameter was found to be increased overall in several retinal vascular plexuses in SWS patients compared to controls, reaching statistical significance (*p* < 0.05 *) in the deep vascular complex. **Conclusions:** The findings from our analysis highlight the potential role of OCTA in predicting the progression or worsening of ocular diseases over time. The introduction of new assessment parameters offers additional perspectives in evaluating ocular health. Since this examination allows for the detection of changes in the morphology and density of blood vessels as revealed by OCTA, these changes can be correlated with disease progression and the effectiveness of therapies.

## 1. Introduction

Sturge–Weber syndrome (SWS) is classified among phakomatoses, a group of disorders characterized by congenital hamartomas [1,2]. Its incidence is estimated at 1 in 20,000–50,000 live births, with equal distribution between sexes, and it typically arises sporadically without hereditary transmission [3,4]. Clinical features include facial capillary malformations (port-wine stain, PWS), leptomeningeal capillary-venous malformations, and ocular abnormalities [5]. PWS, also known as capillary malformation, is the most common cutaneous manifestation and generally involves the forehead and upper eyelid, following the trigeminal dermatome distribution, although recent studies suggest this correlation may not always be strict [6,7,8,9]. Lesions may extend bilaterally or to mucosal areas, becoming darker and thicker with age. Pulsed dye laser remains the mainstay of treatment, requiring repeated sessions for optimal cosmetic outcomes [10,11].

SWS is frequently associated with ipsilateral involvement of the eye, brain, or both. Ocular involvement is frequent and may affect both the anterior and posterior segment. The most common ocular manifestations are glaucoma, which may cause optic nerve damage and vision loss if untreated, and choroidal hemangioma, which can lead to retinal detachment and severe visual impairment. Other vascular anomalies include enlargement of the choroidal plexus, venous malformations, and large deep draining vessels [12].

**Figure 1:**
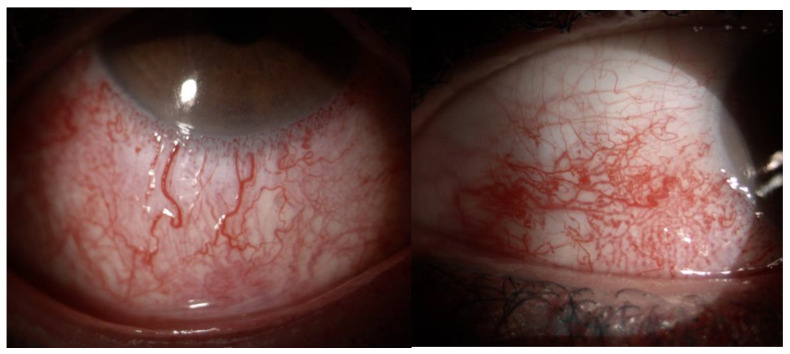
Ampulliform dilations of the conjunctival vessels and visibility of the episcleral venous plexus. Anterior segment examination shows marked vascular abnormalities involving the conjunctival and episcleral circulation. The conjunctival vessels appear dilated, with characteristic ampulliform enlargements along their course. In addition, the episcleral venous plexus is clearly visible through the overlying conjunctiva, indicating venous congestion and impaired outflow.

Examination of the anterior segment typically reveals a dense episcleral venous plexus and ampulliform dilations of the conjunctival vessels, see Figure 1 [13].

Glaucoma represents the most common ocular manifestation, affecting 30–70% of patients [14]. It shows a bimodal distribution, with an early-onset form resembling congenital glaucoma and a late-onset form emerging in adolescence, often bilateral and linked to episcleral venous abnormalities [15,16,17,18,19]. The main mechanisms include developmental defects of the iridocorneal angle and elevated episcleral venous pressure [17,18,19,20,21,22,23].

In juvenile SWS, trabecular meshwork changes resembling primary open-angle glaucoma have been reported, likely linked to vascular malformations and elevated episcleral venous pressure [24,25]. Secondary angle-closure glaucoma, though rare, may occur due to retinal detachment or iris rubeosis [17,26]. Given the lifetime risk of up to 70% [15,16,24,25,27], regular ophthalmologic monitoring is essential to prevent optic nerve damage and vision loss [28].

**Figure 2:**
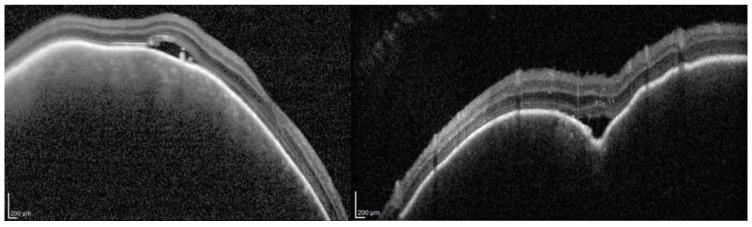
Structural OCT of a choroidal hemangioma associated with subretinal fluid. Structural OCT shows a hyporeflective, dome-shaped lesion at the choroidal level, consistent with a circumscribed choroidal hemangioma. The lesion causes a regular protrusion toward the overlying retina. Hyporeflective subretinal fluid is clearly visible, interposed between the neurosensory retina and the retinal pigment epithelium (RPE), resulting in a modest elevation of the neurosensory retina. The RPE appears slightly elevated and discontinuous overlying the mass. No evidence of neovascularization is detected.

Choroidal hemangiomas are the second most common ocular finding, occurring in about 50% of patients [29]. They may be circumscribed or diffuse and can cause refractive errors, amblyopia, or retinal detachment [29,30,31]. Diagnosis relies on clinical examination and multimodal imaging [32,33,34,35,36], including OCT, fluorescein angiography, indocyanine green angiography [37,38,39,40,41,42,43,44], and enhanced depth imaging OCT, which reveal choroidal thickening and vascular anomalies (Figure 2) [45,46,47,48,49,50,51].

Given the wide spectrum of ocular vascular involvement in SWS, early recognition and close monitoring are essential to prevent vision-threatening complications. Optical coherence tomography angiography (OCTA) has emerged as a non-invasive and highly informative tool for evaluating retinal and choroidal microvasculature in these patients [52,53,54,55,56].

Traditionally, OCTA studies have categorized the retinal vasculature into two main complexes: the superficial vascular complex (SVC) and the deep vascular complex (DVC). However, histological evidence indicates that the human macula actually comprises three distinct retinal plexuses: the superficial vascular plexus (SVP), the intermediate capillary plexus (ICP), and the deep capillary plexus (DCP) [57,58]. In addition, a radial peripapillary capillary plexus (RPCP) has been identified in the peripapillary region, running parallel to the axons of the nerve fiber layer (NFL) [58]. Functionally, the SVC includes both the SVP and RPCP, while the DVC consists of the ICP and DCP. These plexuses are interconnected and converge near the foveal avascular zone (FAZ), thereby defining its boundaries [59,60].

The endpoint of our study was to investigate retinal and choroidal vascularization in patients with SWS using OCT angiography, as compared to controls.

## 2. Materials and Methods

Between April 2022 and April 2024, 15 patients with Sturge–Weber syndrome and 15 healthy controls, with an average age of 35 years (ranging from 8 to 58 years), age and sex matched (8 females and 7 males in each group), were consecutively enrolled at the Department of Ophthalmology, Borgo Roma Hospital, University Hospital of Verona, Verona, Italy.

The study protocol was previously approved by the local Ethics Committee. Informed consent to participate have been obtained from the patients or their parent and/or legal guardian. All data were collected anonymously and following the ethical standards of the Declaration of Helsinki.

Inclusion criteria for the study group were being affected by Sturge–Weber syndrome (SWS), consenting to undergo ophthalmological examination and OCT-angiography (OCTA), and demonstrating sufficient cooperation for the execution of the OCTA examination. Exclusion criteria for the study group were refusal or inability to provide informed consent, poor cooperation or inability to complete the OCTA protocol, and the presence of any concurrent ocular or systemic condition unrelated to SWS that could affect retinal or choroidal vasculature.

Inclusion criteria for the control group were absence of any systemic or ocular pathology, absence of refractive errors exceeding SER > 3 diopters, no history of ocular treatments, consenting to undergo ophthalmological examination and OCTA, and demonstrating sufficient cooperation for the execution of the OCTA examination. Exclusion criteria for controls were any systemic or ocular disease, refractive errors beyond ±3 diopters of spherical equivalent, prior ocular surgery or treatment, insufficient cooperation, or inability to provide informed consent.

### 2.1. Ophthalmological Examinations

All patients underwent a comprehensive ophthalmological examination, which included visual acuity measurement, intraocular pressure (IOP) assessment, and fundoscopic evaluation. Specific imaging techniques were employed to assess retinal structure and vascular architecture. This included the use of structural Optical Coherence Tomography (OCT) and Optical Coherence Tomography Angiography (OCTA), both of which are non-invasive imaging techniques that provide high-resolution cross-sectional images of retinal layers and allow for the visualization of the retinal and choroidal vasculature, respectively.

Among SWS patients, 9 out of 15 were amblyopic and 8 out of 15 had glaucoma; 8 out of 15 presented with vascular malformation. Control group patients all demonstrated normal visual acuity in both eyes, a spherical equivalent refraction (SER) not exceeding 3 diopters, normal intraocular pressure, normal findings on anterior and posterior segment examination, and no history of ocular treatments.

### 2.2. Imaging Device and Settings

For imaging acquisition, we used the Heidelberg Spectralis OCT device (Heidelberg Engineering, Germany) with software version 6.12.

This system utilizes spectral-domain OCT (SD-OCT) technology, which captures images at high speed and with excellent resolution, making it ideal for analyzing subtle microvascular changes in both the retina and choroid. To ensure consistency and reliability in data collection, all imaging sessions were conducted by the same experienced ophthalmologists (R.L. and L.P.). The OCT and OCTA scans were centered on the macular region with dimensions of 3 × 3 mm. These settings were selected in High-Speed (HS) mode, particularly for patients with severe visual impairment or difficulty in maintaining fixation, which is often seen in patients with significant amblyopia or nystagmus. HS mode provides faster scanning while maintaining sufficient image quality. The images were adjusted for corneal curvature, fixation and patient positioning for patient age and level of cooperation. All accepted OCTA images showed Q-score C 20, and those of poor quality or with segmentation errors were excluded from this analysis.

OCT and OCTA images were acquired with participants seated and positioned facing the SD-OCT device, following the OSCAR-IB criteria [12] and the APOSTEL 2.0 recommendations [13]. During the scan, subjects were instructed to maintain fixation on a designated light target. The number of scans was consistent across all participants, and poor-quality images were excluded from the analysis.

High-resolution images with a lateral resolution of 5.7 μm/pix are provided by the SPECTRALIS^®^ OCT Angiography (OCTA) Module (Heidelberg Engineering, Germany). Joined with the accuracy of TruTrack Active Eye Tracking, this tool allows for detailed visualization of intricate capillary networks. With an axial resolution of 3.9 μm/pixel, it enables the segmentation of all four histologically validated retinal vascular plexuses.

Since in our study we analyzed only the macular region we took into account only SVP as far as SVC is concerned.

Finally, avascular complex (AC) extends from the outer nuclear layer (ONL) till Bruch’s membrane (BM) and, as the name itself suggests, no vascular plexuses are found at this level.

### 2.3. Image Processing and Analysis

Fiji software (ImageJ, version 2.9.0, Computer software, Max Planck Institute of Molecular Cell Biology and Genetics, Dresden, Germany) was employed for post-processing of the OCTA images. The raw images were initially binarized to distinguish the blood vessels from the surrounding tissue, thereby facilitating the quantification of vascular parameters. The vessel density index was calculated as the ratio between the white pixels (representing blood vessels) and the total number of pixels within the image area. This approach allows for a more objective evaluation of vascular density.

**Figure 3:**
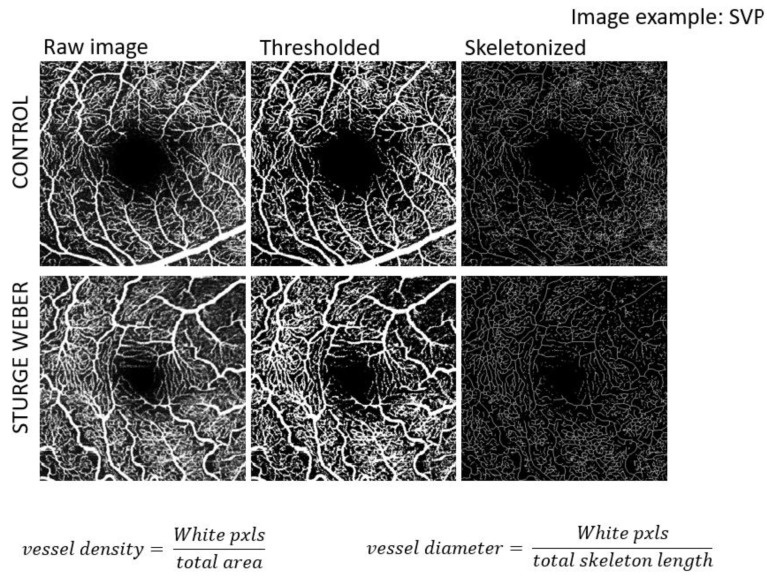
Image processing: threshold, skeletonization. The OCTA images underwent a pre-processing pipeline designed to optimize vascular quantification. First, Gaussian filtering was applied to suppress background noise while preserving vessel edges. Subsequently, the en face OCTA slabs were binarized into black-and-white images, with white pixels representing the vasculature. The binarized vasculature was then skeletonized, reducing the vessels to one-pixel-wide centerlines to allow for precise geometric and length estimation. From these steps, two primary quantitative metrics were derived within the region of interest (ROI). The vessel density was calculated as the ratio between the number of white pixels from the thresholded image and the total area, whereas the vessel diameter was calculated as the ratio between the number of white pixels from the thresholded image and the total skeleton length obtained from the skeletonized image.

To improve image quality before analysis, each image underwent a Gaussian filter, to reduce noise and enhance the visibility of the vascular structures. The binarized images were subsequently skeletonized to create a one-pixel-wide representation of the blood vessels, allowing for more precise measurements of vessel length and structure. The vessel diameter was then determined by calculating the ratio of white pixels to the total length of the skeletonized image, providing a measure of the average vessel thickness in the selected region, Figure 3.

### 2.4. Statistical Analysis

Microsoft Excel (Microsoft Corporation, Redmond, WA, USA) was used to analyze the quantitative data. Statistical significance was assessed using Student’s *t*-test and a *p*-value < 0.05 was considered statistically significant, suggesting that the differences observed between the Sturge–Weber syndrome patients and the control group were unlikely to have occurred by chance.

### 2.5. Limitations of Current OCTA Technology

It is important to note that, as of the time of this study, the parameters we analyzed—such as vessel delineation, binarization, and skeletonization—cannot be automatically detected using commercially available OCTA software. This limitation necessitated the use of manual image processing techniques and post-acquisition software to extract the desired vascular features from the OCTA scans.

## 3. Results

The analysis of the images revealed that the vascular density of the Deep Capillary Plexus (DCP) was significantly increased (*p* < 0.05 *) in patients with SWS compared to healthy controls (Table 1).

The vessel diameter shows differences across the different retinal vascular plexuses (SVC, SVP, DVC) in patients with SWS compared to controls, reaching statistical significance (*p* < 0.05) at the level of the Deep Vascular Complex (DVC). The results show also a highly significant *p* value for vessel diameter in the DCP in SWS patients compared to controls (*p* = 0.00000001) (Table 2).

**Figure 4:**
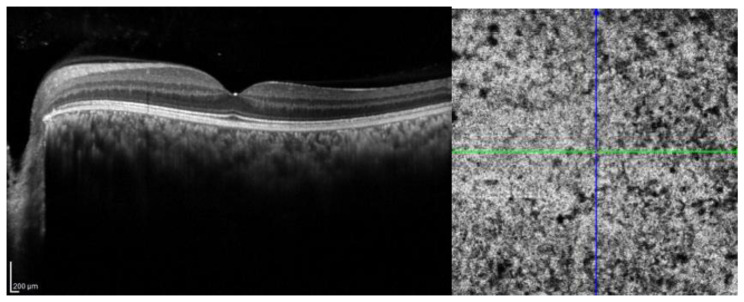
Structural OCT of a thickened choroid in a patient of our series and the typical appearance of “worm sack” on OCT angiography. Structural OCT demonstrates diffuse choroidal thickening, with increased subretinal choroidal thickness and reduced distinction between the individual vascular layers. The neurosensory retina appears relatively preserved, without marked atrophy, but shows a slight elevation corresponding to areas of increased hyperreflectivity. OCT angiography (OCTA) reveals the characteristic “worm sack” appearance, defined by an irregular network of abnormal, tortuous vessels arranged in a disorganized fashion, reflecting profound vascular alterations and disruption of the normal choroidal microvascular architecture.

**Figure 5:**
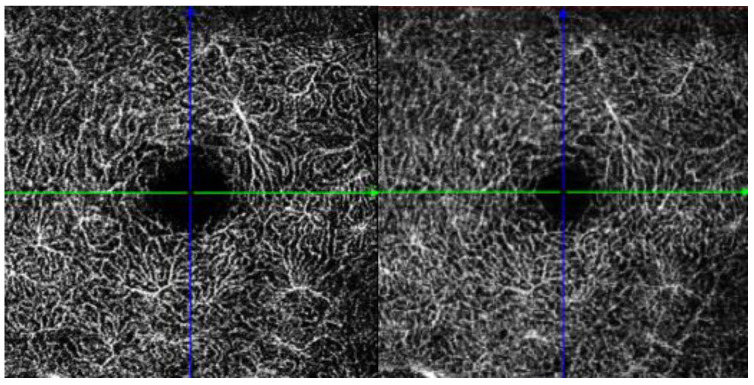
OCT angiography of Deep Capillary Plexus (DCP) and Deep Vascular Complex (DVC) of the same patient above. At the DCP level, the vascular meshwork appears irregular with focal zones of capillary dropout alternating with areas of dilated and tortuous capillaries, indicating localized ischemia and abnormal compensatory remodeling. At the level of the DVC, a vascular disorganization is also appreciable, with convoluted and densely intertwined vascular loops merging into a chaotic configuration resembling a ‘worm sack’. These alterations reflect the pathological vascular remodeling characteristic of Sturge–Weber syndrome and highlight the added value of OCTA in capturing subtle microvascular changes not evident on structural OCT alone.

To assess whether the statistically significant difference observed between the study group and the control group was also clinically significant, we calculated the effect size using Hedges’s g, which is more suitable for small sample sizes. We found that the DCP vessel density Hedges’s g is 0.68, indicating a medium/high effect, the DVC vessel diameter Hedges’s g is 0.68, and the DCP vessel diameter Hedges’s g of 4.89 corresponds to a large effect (Figure 4 and Figure 5).

## 4. Discussion

To date, no studies have systematically investigated retinal microvasculature in SWS patients compared with healthy controls. The retina, being easily accessible to non-invasive imaging and suitable for repeated evaluations, represents an optimal site to monitor structural changes and guide clinical decisions in these patients. In the literature, only one case report has documented vascular dilation at the DCP in SWS [61].

Our pilot study demonstrates a significant increase in vascular density at the DCP and in vessel diameter at the DVC in SWS cases compared to controls (*p* < 0.05 *). The results also show a highly significant difference in vessel diameter at the DCP in SWS patients compared to controls (*p* = 0.00000001 *) (Table 2). This result, which remains statistically robust even with the relatively small sample size, indicates a marked microvascular alteration specifically affecting the deepest retinal plexus. The DCP, located at the outer retina and adjacent to the avascular zone, is particularly vulnerable to hemodynamic changes and impaired vascular remodeling, which may explain the pronounced reduction observed. The extremely low *p*-value reflects not only the consistency of the difference across the cohort, but also the small standard deviation in the SWS group, suggesting a homogeneous pathological pattern. Importantly, unlike previous studies in the literature, our work provides quantitative figures specifically for the DCP and demonstrates that not only vascular density but also vessel diameter can undergo clinically relevant changes in SWS [62,63,64]. Taken together, these findings highlight the DCP as a critical site of vascular involvement in SWS, potentially serving as a sensitive biomarker for disease-related microvascular changes.

Although we cannot draw definitive conclusions on the relationship between vascular malformations and glaucoma given the small cohort, we observed that glaucoma tends to be more severe in patients with extensive vascular involvement. OCTA, by providing high-resolution, non-invasive visualization of retinal and choroidal microvasculature, represents a valuable tool for detecting vascular changes and informing targeted management [62] using biomarkers that assess vascular density in the choroid, monitoring choroidal health status, and predicting the risk of developing certain pathological conditions [63]. Previous studies have shown that OCTA may reveal episcleral vascular alterations correlating with IOP and glaucoma risk [64], and characterize choroidal hemangiomas by analyzing vascular density and flow [45,65].

An important advantage of OCTA is its reproducibility, allowing reliable monitoring of vascular modifications over time, especially in glaucoma associated with SWS [65]. The technology’s ability to produce consistent, high-resolution scans of retinal and choroidal microvasculature is especially beneficial in SWS, where vascular anomalies may change over time. Studies have confirmed that OCTA measurements, especially those evaluating vessel density and morphology [65], maintain high reproducibility across sessions, reinforcing its value in long-term follow-up. This consistency is crucial in managing glaucomatous changes in SWS patients, where precise tracking of vascular modifications supports timely and informed clinical decisions.

Evidence from other vascular diseases, such as diabetic macular edema, has shown that OCTA-derived biomarkers may predict treatment response [66]. While this association has not yet been studied in SWS [62], our preliminary data suggest that OCTA could provide prognostic biomarkers in this context, pending confirmation by larger studies [67,68,69,70,71].

The main limitation of our study is the small sample size, a reflection of the rarity of SWS (1 in 20,000–50,000 live births worldwide). Additional challenges include poor cooperation in some patients due to neurological impairment or nystagmus, as well as the use of manual image processing, which may affect reproducibility. Furthermore, the cross-sectional, single-center design limits generalizability and prevents assessment of longitudinal changes. Future multicenter studies with larger cohorts and automated OCTA analysis are needed to validate our findings and establish standardized protocols. Another limitation of this study is the use of manual image processing techniques (binarization and skeletonization), which may affect reproducibility, although all analyses were performed by a single operator to reduce variability. However, the possibility of validating the proposed analysis methodology with additional studies suggests that our pipeline could, in the future, lend itself to automation. The integration of deep learning algorithms for segmentation, quality control, and cross-vendor standardization could further enhance reproducibility and clinical applicability of OCTA analyses.

Our preliminary results indicate that OCTA is a promising tool for evaluating vascular anomalies in SWS. Further multicenter studies would allow for confirmation of its reproducibility and clinical utility across diverse patient populations and clinical settings. Such validation would help establish standardized OCTA protocols and diagnostic criteria, ensuring that OCTA measurements are consistently accurate and applicable in clinical practice for SWS patients

Nevertheless, our results provide innovative preliminary evidence of vascular alterations at both the DCP and DVC in SWS, supporting the role of OCTA as a promising tool for diagnosis, monitoring, and potentially guiding treatment in this rare syndrome.

## 5. Conclusions

OCTA is emerging as a valuable non-invasive tool for detecting retinal and choroidal microvascular alterations in SWS, with potential biomarkers such as changes in vascular density and vessel caliber that may support early diagnosis, monitoring of disease progression, and treatment response. Importantly, our proposed analysis pipeline, although currently manual, could in the future be automated through deep learning–based segmentation, quality control, and cross-vendor standardization, thereby improving reproducibility and broadening the clinical applicability of OCTA in this rare condition.

## Figures and Tables

**Table 1:** Comparison of retinal vascular density between Sturge–Weber patients (SWS) and controls, showing significantly higher vessel density in the Deep Capillary Plexus (DCP) in SWS (*p* < 0.05). Superficial Vascular Complex (SVC), Superficial Vascular Plexus (SVP), Deep Vascular Complex (DVC), Intermediate Capillary Plexus (ICP), Whole Retina (RET) Slab, Full-Thickness Slab (FULL). Vessel Density is dimensionless (reported as %).

Layer	CTL Mean ± SD	SWS Mean ± SD	*p*-Value
SVC	0.2584 ± 0.0530	0.2385 ± 0.0611	0.27
SVP	0.3400 ± 0.0663	0.3173 ± 0.0720	0.29
DVC	0.2521 ± 0.0454	0.2644 ± 0.0608	0.43
ICP	0.2028 ± 0.0434	0.2079 ± 0.0508	0.72
DCP	0.2180 ± 0.0591	0.2591 ± 0.0612	0.03
AVASCULAR	0.2460 ± 0.0369	0.2457 ± 0.0467	0.98
CHORIOCAPILLARIS	0.3832 ± 0.0910	0.3126 ± 0.1012	0.16
CHOROID	0.3705 ± 0.0525	0.3530 ± 0.1166	0.48
RET	0.2320 ± 0.0506	0.2343 ± 0.0965	0.96
FULL	0.3101 ± 0.0454	0.3114 ± 0.0935	0.94

**Table 2:** Comparison of the vessel diameter of the retinal vascular plexuses on OCT angiography in Sturge–Weber patients and controls. Quantitative OCTA analysis demonstrated that vessel diameter showed a tendency to increase across multiple retinal vascular plexuses, including the superficial vascular complex (SVC), superficial vascular plexus (SVP), and deep vascular complex (DVC). Among these, the difference reached statistical significance at the level of the DVC (*p* < 0.05). Intermediate Capillary Plexus (ICP), Whole Retina (RET) Slab, Full-Thickness Slab (FULL). Vessel diameter was quantified in pixels, with an optional conversion into micrometers (µm) using a scale factor of 5.7 µm/pixel (3 × 3 mm protocol). The µm estimates were calculated as *value [pixels] × 5.7*, without applying axial-length correction. Accordingly, the Vessel Density Parameter (VDP) was defined as the ratio between the number of vessel pixels (N_vessel-pixels_) and the length of the vascular skeleton (L_skeleton_), expressed in pixels.

Layer	CTL Mean ± SD	SWS Mean ± SD	*p*-Value
SVC	4.0340 ± 0.5318	4.1998 ± 0.5337	0.34
SVP	3.9783 ± 0.3928	4.0895 ± 0.4155	0.41
DVC	3.4600 ± 0.1835	3.6589 ± 0.3883	0.02
ICP	3.5068 ± 0.1654	3.4719 ± 0.1944	0.51
DCP	3.5160 ± 0.1997	2.7260 ± 0.0643	0.00000001
AVASCULAR	No skeletonization	-	-
CHORIOCAPILLARIS	No skeletonization	-	-
CHOROID	No skeletonization	-	-
RET	3.1523 ± 0.1878	3.1334 ± 0.1958	0.75
FULL	4.2806 ± 0.3945	4.5014 ± 0.5621	0.13

## Data Availability

Data are available, upon request.
